# Development of the Fatigue Risk Assessment and Management in High-Risk Environments (FRAME) Survey: A Participatory Approach

**DOI:** 10.3390/ijerph16040522

**Published:** 2019-02-13

**Authors:** Ashley E. Shortz, Ranjana K. Mehta, S. Camille Peres, Mark E. Benden, Qi Zheng

**Affiliations:** 1 Department of Environmental and Occupational Health, Texas A&M University, College Station, TX 77843, USA; aeshortz@tamu.edu (A.E.S.); peres@tamu.edu (S.C.P.); mbenden@sph.tamhsc.edu (M.E.B.); 2 Department of Industrial and Systems Engineering, Texas A&M University, College Station, TX 77843, USA; 3 Department of Epidemiology and Biostatistics, Texas A&M University, College Station, TX 77843, USA; qzheng@sph.tamhsc.edu

**Keywords:** oil and gas, overexertion, sleep, shift, workload, questionnaire

## Abstract

Existing risk assessment tools are not effective or sustainable in identifying Oil and Gas Extraction (OGE) workers at high risk of fatigue-related injuries or incidents. We developed a comprehensive Fatigue Risk Assessment and Management in high-risk Environments (FRAME) survey through an industry-academic participatory approach. The FRAME survey was developed through: (1) systematic gathering of existing fatigue scales; (2) refining the inventory using the Delphi Consensus technique; and (3) further refinement through employee/worker focus groups. The participatory approach resulted in a final FRAME survey across four fatigue dimensions—sleep, shiftwork, physical, and mental fatigue, and was composed of 26 items. The FRAME survey was founded on occupational fatigue science and refined and tailored to the OGE industry, through rigorous industry stakeholder input, for safer, effective, practical, and sustainable fatigue assessment and management efforts.

## 1. Introduction

Fatigue plays a vast role in all industries in terms of performance, safety, and productivity and is continually ranked among the top five human factors issues year after year [1]. It is estimated that fatigue costs more than $18 billion per year in lost productivity alone, of which 84% is due to reduced performance at work, rather than absenteeism [2]. Fatigue in oil and gas operations specifically can have detrimental and catastrophic effects. An example is the BP Texas City Incident of 2005, which resulted in the deaths of 15 workers, 180 injuries, and a loss of at least $1.5 billion [3]. Fatigue was identified as a contributing factor as some operators had been working 12-h shifts for as many as 29 consecutive days. Another major incident was the Piper Alpha oil platform disaster that resulted in the deaths of 167 workers [4]. It was determined that poor communication at the shift turnover was a contributing factor. Currently, due to the lack of consistent, sufficient, and effective incident reporting methods, it is difficult to estimate the extent to which fatigue has contributed to oil and gas extraction (OGE) incidents and injuries [5,6,7]. However, it is estimated that up to 80% of industrial accidents and incidents are due to human errors, for which fatigue was often partly responsible [5].

While the OGE sector is one of the most regulated sectors in terms of safety standards and regulations [8], fatalities in OGE are still alarmingly high, especially when compared to all other U.S. workers. The fatalities rate in this sector is about seven times greater [9]. This could be due to the dynamic environment surrounding OGE operations, including high physical and mental workload, coupled with long work periods and shiftwork and prolonged social isolation [10]. The disproportionate fatality rate in this industry could be due to the complex combination and interaction of these factors contributing to high levels of fatigue. For example, OGE workers are typically working in remote locations for a 2–3 weeks’ deployment and these locations are among the most demanding in terms of the technology used in daily operations and workforce demands [10]. Usually work schedules in OGE operations are 12-h periods in two shifts (day and night). While it has been established that 12-h shifts are not necessarily unfavorable, when they are coupled with long work weeks and night work, which are common practices in OGE operations, concomitant adverse events have been observed [11]. In addition to long work periods and shiftwork, OGE workers, especially drillers, are confronted with extreme cognitive demands. Drillers are responsible for the day-to-day operations on the rig. This includes monitoring and maintaining well control (i.e., to avoid blowout), organizing and leading work on the rig floor, as well as ensuring safety of the team [12]. Such high cognitive demands, paired with long work days and rotating schedules could potentially compound fatigue levels, thus increasing the risk of an adverse event. 

Due to the multidimensional nature of fatigue, effective fatigue assessment is a critical challenge, particularly when considering the complex OGE working environment. While there is no gold standard for measuring comprehensive fatigue, several objective and subjective fatigue assessment methods exist that have been employed in different occupational sectors. The method for assessing fatigue is typically tailored to the environment and setting in which fatigue is being evaluated and can be considered as research-oriented (i.e., neuroimaging techniques and electrocardiogram) [13,14] and/or practice oriented in nature (i.e., performance test and subjective assessments) [15,16]. For example, within the transportation sector, there is an abundance of literature that demonstrates utility of different assessment methods to measure driver fatigue. Traditional lab-based studies have used methods that include brain activity via electroencephalography (EEG), eye movements using electrooculography, cardiovascular responses, biochemical markers, psychomotor tests, and subjective responses [17,18,19], while onboard devices exist that use EEG, which monitor and detect changes in drivers’ states [20]. In the aviation sector, there is a trend of using performance tests such as the psychomotor vigilance test as a primary measure of fatigue rather than traditional lab-based methods [21], whereas subjective surveys are typically used within the healthcare industry [22,23]. 

A recent study employed real-time physiological monitoring in order to assess shiftwork in a 3-week offshore operation [24,25]. While valuable information was gained from physiological monitoring, the authors argued that this fatigue assessment approach was not sustainable or feasible for the OGE environment. Due to the hazardous and volatile environment of offshore operations, introducing sensors into this environment may create additional safety risks. This presents a challenge of identifying workers with high-risk fatigue levels. Without effective assessment methods, fatigue management practices that are targeted and effective in reducing fatigue-related incidents in the OGE industry are likely to be ineffective.

A potential safe and sustainable solution could be a non-intrusive, industry-specific subjective assessments tool for assessing fatigue in high-risk environments. Indeed, research within the OGE domain has traditionally assessed fatigue using subjective surveys and questionnaires [26,27,28,29,30]. However, the question remains whether these subjective research tools can be practically adopted in everyday OGE operations to assess worker fatigue. Mehta et al. [25] augmented physiological monitoring in their offshore shiftwork investigation with existing subjective methods to assess fatigue. The surveys included some of the more occupationally used fatigue scales, such as the Swedish Occupation Fatigue Inventory and the Occupational Fatigue Exhaustion Recovery [26,27,28]. They found that none of these surveys were comprehensive, relevant, and feasible for the OGE workforce. For example, compliance for completing these surveys declined over time due to the time-intensive nature of completing the surveys. On average, it took workers approximately 45 min to complete all the surveys used in this study. Additionally, the workers reported that some of the questions were not clear and they had trouble understanding certain terms (e.g., some were unsure what “palpitations” meant). Because the SOFI was developed for the healthcare domain, the descriptions included medical terminology that nurses and caregivers are familiar with. As such, Mehta et al. [25] highlighted a critical gap in OGE fatigue assessment research and practice. Thus, feasible and sustainable fatigue assessment surveys need to be developed for the OGE sector that address user needs and education levels or utilize stakeholder feedback in OGE-specific hazardous work environment. 

One methodology that holds strong potential to address tool development disconnect between research and practice is the Delphi consensus technique. The Delphi consensus technique is an iterative process that typically involves three to four rounds of questionnaires until consensus is reached regarding a specific topic. This technique has been employed successfully toward the development of human factors risk quantification in oil and gas drilling operations [31], fatigue surveys [32], as well as medical checklists used for safe surgical practices [33,34], and forecasting and issue identification, and framework development [35,36]. Since the Delphi consensus technique has been successfully applied in the OGE sector for human factors and in the use of other fatigue survey development, this technique provides a method that would be ideal for the development of an OGE specific fatigue survey.

The purpose of this study was to develop a comprehensive fatigue assessment tool specific for OGE operations utilizing the Delphi Consensus technique with participation from several OGE industry stakeholder groups, such as safety managers, consultants, researchers, and workers. To our knowledge, this was one of the first collaborations integrating user feedback from the OGE stakeholders and workers as well as occupational health and safety researchers. This effort will increase the likelihood that the new fatigue assessment tool will be adopted by the OGE industry, thus increasing its sustainability [37]. 

## 2. Materials and Methods 

This study adopted a qualitative approach to develop a fatigue assessment inventory. A critical component in the development of this method was the integration of end user, i.e., OGE industry, inputs and expertise throughout the development process. This was completed in three phases. Phase 1 included a systematic gathering of existing fatigue survey based on predetermined criteria to develop a preliminary fatigue inventory. Phase 2 included the refinement of the inventory for content validation, conciseness, and relevance to OGE operations through industry stakeholders (i.e., safety managers from major OGE companies) and health and safety researchers (i.e., current doctoral students, post-doctoral researchers, and faculty from Department of Environmental and Occupational Health at Texas A&M University) using the Delphi consensus technique. Phase 3 involved further refinement of the inventory by OGE workers, who are the intended end-users. The inventory was refined for language and relevance to daily OGE work in this phase.

### 2.1. Fatigue Inventory Development (Phase 1)

Before the development of any assessment technique, the construct needs to be clearly defined and operationalized for the specific domain. In this study, fatigue in the OGE environment was operationally defined as a physiological state of reduced mental or physical performance capability resulting from sleep loss, disrupted circadian phase, and high workload (physical and mental). This definition was borrowed from the comprehensive definition of fatigue in aviation [38]. 

Potential items under consideration for developing the fatigue inventory were obtained from a systematic gathering of articles published from 1970 to 2016 and indexed in the Scopus database. Scopus was chosen for its thorough coverage of more than 22,800 titles with subject areas ranging from social sciences (psychology, economics, and business: 8698 titles), health sciences (100% MEDLINE, nursing, and dentistry: 7133 titles), physical sciences (chemistry, engineering, and physics: 7441 titles) and life sciences (neuroscience, pharmacology, and biology: 4601 articles). The criteria for inclusion was determined as: (1) ability to identify distinct fatigue symptoms, (2) applicability to health workforce, (3) sensitivity to a range of fatigue levels, and 4) English language. The search was completed in April 2016. Search terms included: fatigue, physical demand, cognitive demand, psychosocial demand, stress, sleep, shiftwork, staff, adult, worker, employee, healthy, scale, assessment, and survey.

### 2.2. Fatigue Inventory Refinement (Phases 2 and 3)

Phase 2 included the refinement of the preliminary inventory for content validation, brevity, and relevance to OGE operations using two Delphi Consensus panels [39]. The size of Delphi panels varies widely. However, most studies using the Delphi Consensus method use panels of 15 to 35 people [40]. The Delphi consensus method used for this study was composed of 13 OGE stakeholders, with representation from researchers, consultants, and practitioners. Upon informed consent, the Delphi panel was presented with the preliminary fatigue inventory and instructed to exclude items based on the following criteria: (1) too general at describing a symptom of fatigue, (2) redundant of previous item, and (3) not relevant to OGE operation. Each item was vetted and discussed and then voted on whether to keep or remove the item. Items with a score of less than 80% consensus were removed. In Phase 3, focus groups were conducted with OGE workers, who are the intended end-user. OGE workers were provided with the refined inventory from the two Delphi consensus conducted in Phase 2. The inventory was further refined for language and relevance to OGE work. 

### 2.3. Participants

Participants were recruited for Phases 2 and 3. Participant demographics are shown in Table 1. For Phase 2, OGE stakeholders, health and safety researchers and practitioners, were recruited to participate in the Delphi consensus study to refine the preliminary fatigue inventory. Industry stakeholders (from major energy companies based in Houston, TX; 61,000–75,000 employees) were invited for the Mary Kay O’Connor Process Safety Center at Texas A&M University. For Phase 3, one focus group was formed, with OGE workers across different job categories from a medium-sized (Houston, TX; 5000 employees) well-servicing company. Their role was to further refine inventory based on the language and relevance to OGE operations. Participants across both phases provided their informed consent approved through the Texas A&M Institutional Review Board (IRB2016-0676M).

## 3. Results

### 3.1. Fatigue Inventory Development (Phase 1)

**Figure 1:**
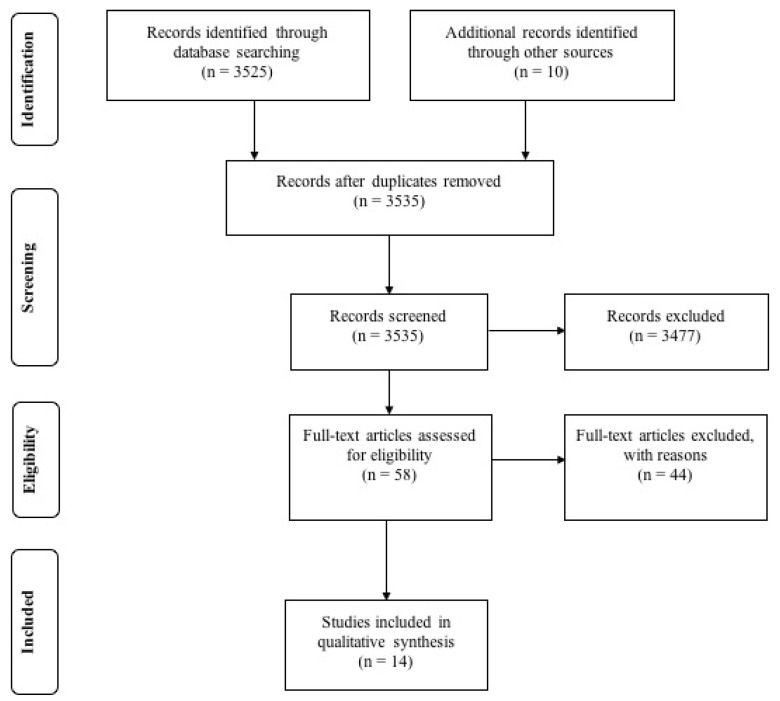
Flowchart of articles gathered.

Figure 1 illustrates the flowchart for number of articles identified in each of the steps described above. Ultimately, 14 peer-reviewed articles were included and reviewed, which yielded 12 widely used surveys, scales, or questionnaires (2 surveys were duplicated) [12,23,32,41,42,43,44,45,46,47,48,49]. This resulted in 230 question items. These questions were directly used with their accompanying instructions and scales in Phase 2.

### 3.2. Fatigue Inventory Refinement (Phase 2)

This phase was completed in two steps utilizing two different Delphi Consensus panels. The first panel (Table 1) was provided the initial inventory, created in Phase 1. The panel was instructed to refine the inventory by improving grammar and language, and by eliminating redundant items. Two hundred and thirty items were discussed and the consensus technique led to a reduced 50-item inventory. Some examples of items that were removed due to vagueness of description included: (1) “*I feel tired*” (2) “*Thinking required effort*” and (3) “*I feel weak*,” Another example for an item that was removed due to redundancy (e.g., “*My thoughts easily wonder*”) was similar to a previous item (e.g., “*I have trouble concentrating*”). Items such as “*I don’t do much during the day*” and “*I think I do a lot during the day*” were removed as the panel argued that truthful responses will be difficult to obtain as responders might be afraid to lose their job or human resources might interfere. An example of an item that was included after rewording the description was “*I use my days off recovering from work*” from the original “*I use a lot of my spare time recovering from work*”. Another example was “*Vision is blurred*” was changed to “*Vision is blurred or distorted*.” This was revised to combine two items on the preliminary inventory. 

The panel was also required to reach consensus regarding how far the worker need to reflect (e.g., right now, past 24 h, or past week) to provide their responses on the fatigue symptoms. All panel members felt that a time frame (i.e., right now vs. previous week vs. three months ago) for the instructions of the inventory needed to be established. A majority of the panelists stated that asking “*right now*” would not only reflect the most accurate symptom response from the worker, but also provide supervisors real-time valuable feedback from the workers necessary for implementing quick fatigue mitigation strategies (e.g., reallocation/rescheduling of personnel and work tasks).

In step 2, 5 OGE stakeholders refined the inventory for OGE relevance and/or utility of the responses on the items to guide management strategies. This reduced the inventory to 38 items. However, the panel added a question they felt was important regarding assessing cognitive fatigue. The added item was “*Fatigue interferes with my mental functioning*” which was developed from “*Fatigue interferes with my physical functioning.*” Two items were removed based on the panel’s suggestion that responses would not be accurate due to the respondent being afraid of losing job or to the Human Resources tending to interfere. These items included “*I frequently dozed off during break period*” and “*I feel no desire to do anything*.” Some examples of items removed due to redundancy included: (1) “*I can concentrate well*” and (2) “*Having tremors in limbs.*” This brought the total items to 39 items at the end of Phase 2.

The panel gave similar feedback about the importance of inventory instructions. The panel suggested that all items began with the same phrase of either “*To what extent...*” or “*To what degree…*” but “*To what degree…*” was favored. Additionally, a timeframe of “*right now*” or “*during the last shift*” was determined as the most critical and valuable for fatigue management. 

### 3.3. Fatigue Inventory Refinement Through OGE Worker Focus Group (Phase 3) 

Eleven OGE workers refined the inventory for OGE relevance and language. In this phase, all 39 items were kept. Feedback from the focus group was used to reword questions for better comprehension, which is expected to allow workers to accurately respond to questions. The workers agreed that all 39 items were relevant to OGE operations. However, they suggested that a few items be reworded. For example, the item “*To what degree do your joints (e.g., knee or elbow) feel achy?*” was changed to “*To what extent do your joints (e.g., knee or elbow) feel stiff or achy?*” Another example included changing the item “*To what degree do you experience tense muscles?*” to “*To what extent do you experience stiff muscles?*”

Additionally, workers gave their input on the type of scale they would prefer to respond to (i.e., 1 (*none*) to 7 (*a lot*) vs. 0 (*Not at all*)-10 (*Extremely*)). Of the 11 workers, 9 preferred the 0 (*not at all*) to 10 (*extremely*), and the other two did not have any comments regarding the response scale. A majority of the workers stated that it was natural for them to perceive zero as “*nothing*” or “*not at all*”, while 1 meant a little bit or slightly more than zero. Additionally, workers expressed that they preferred “*to what extent…*” rather than “*to what degree…*”. The workers also suggested that having a Spanish version of the final inventory would be critical for implementation and translation to sustainable practice. Over 50% of workers spoke Spanish as their first language. They expressed that while they understood the English version, it would be easier for them to understand and respond accurately and quickly with a translated inventory.

### 3.4. Final Fatigue Inventory (FRAME)

**Figure 2:**
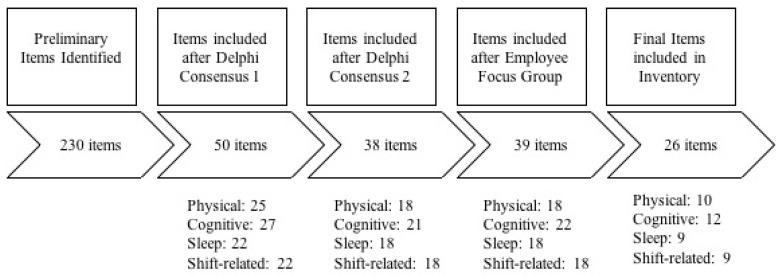
Summary of Inventory Item Reduction.

After health and safety researchers, OGE stakeholders, and OGE workers reviewed the preliminary inventory of 230 items, the final inventory decreased to 39 items. The 39 items were further reviewed by the study research team to verify that all dimensions of fatigue (e.g., physical, mental, sleep, and shift-related) were represented. In this review, the research team identified 13 items that would not offer sufficient value to the organization (e.g., supervisors, safety managers, etc.) for implementing effective fatigue management practices, but that could provide a better understanding of workers’ personal strategies to manage fatigue. Because the original purpose of the fatigue inventory was to help organizations develop effective and sustainable fatigue mitigation strategies, these items were removed to enhance ease of use of the new tool. Examples of the removed items included: (1) “*To what extent do you worry about issues at home making it hard to relax?*” (2) “*To what extent do you use alcohol to help you sleep?*” (3) “*To what extent are you concerned for how long you can keep going at your work?*” and (4) “*To what extent would you work overtime or an extra shift when you are sleepy or fatigue in you needed extra money?*” It should be noted that the mentioned items were discussed extensively in Phase 1 and Phase 2. Participants from both the Delphi Panels and Employee focus groups suggested that these were interesting items and hence were ultimately kept in the inventory, but unsure how responses could be used for fatigue management programs. Ultimately, 26 items were selected for final fatigue inventory (Figure 2). Figure 2 also lists the number of items that were classified within each dimension of fatigue (physical, mental, sleep or shift-related) in each phase. It should be noted that items can be classified as more than one dimension. An example of this is the item “*fatigue interferes with carrying out certain duties and responsibilities.*” This item could be classified as all four dimensions. 

The final fatigue inventory recommended by the research team along with when the question should be asked (pre-shift or post-shift) is shown in Table 2.

## 4. Discussion

The aim of this study was to develop a comprehensive fatigue assessment inventory specific for OGE operations through involvement of OGE stakeholder input. The resulting survey instrument, named Fatigue Risk Assessment for high-Risk Environments (FRAME) survey has a total of 26 items encompassing four dimensions of fatigue. It should be noted that this survey was developed by obtaining input from the OGE industry, and as such its application to other high risk industries, such as mining, need to be evaluated.

A recent study comparing objective and subjective fatigue assessment in offshore work used five different surveys to capture comprehensive operator fatigue over the course of a hitch [25]. An average worker needs approximately 45 min to complete all the surveys as the wordings were not clear difficult to understand. As a result, compliance was low as time progress over the hitch. Moreover, lengthy surveys are at risk of resulting in participant response fatigue, implying that questions asked later in a long survey are typically prone to increased error and untruthful and unreliable responses [50]. Pilot studies have found the FRAME survey, developed here, takes no longer than 10 min to complete, simply due to the reduced number of times [51]. This would allow for compliance to be greater and additionally recovery time between shifts, as operators will not spend a great amount of time completing surveys. Sustained use of the FRAME survey could help to facilitate the successful implementation of effective fatigue management practices outline in the API Recommended Practice 755: Fatigue Risk Management System [52]. 

Fatigue-related incidents have been continually identified as one of the most dangerous risk factors in high-risk industrial setting [27]. When workers were asked to define fatigue, most simply said it’s when they are “tired” or “exhausted”. Fatigue is typically described as tiredness, overexertion and strenuous movements [27]. Overexertion injuries were the third leading cause for days away from work in 2007 behind being struck by an object or caught in object, equipment, or material [53]. Currently, the most common method for assessing physical fatigue in OGE sector is surveys and questionnaires [26,29,30,54]. Regarding physical workload, the FRAME survey has 10 items to gauge physical fatigue, ensuring that this important dimension of fatigue is assessed through the new tool.

Not only does physical workload affect fatigue-related performance and safety outcomes, intense mental workload associated in specific operations, specifically drilling and well control scenarios, can contribute significantly to fatigue development. In both onshore and offshore operations alike, drillers are responsible for maintaining and ensuring rig safety, in addition to performing day-to-day tasks required for rig performance. The day-to-day tasks are outlined by a well operation plan [55]. The well operation plan is an overview of the drilling parameters that must be monitored and maintained. In addition to following the well operation plan, drillers are also responsible for providing well control if necessary. This means that in the case of an adverse event such as a kick or loss of circulation, they are required to intervene to avoid blowout [55]. Performance on daily operations relies heavily on cognitive dexterity [12]. Similar to physical fatigue assessments, the most common method that is currently used to assessing cognitive fatigue in OGE is surveys and questionnaires such as the Swedish Occupational Fatigue Inventory and Work Ability Index [26,27,28]. However, none of these surveys were designed specifically use for OGE operation. Since the FRAME survey was developed with OGE input and consideration, it is better suited than current surveys being used for cognitive fatigue assessment. To assess cognitive fatigue, the FRAME survey has 12 items. It might be argued that workers may tend to respond to some questions based on their awareness rather than their experience (for e.g., question item 18 in Table 2). Future testing (such as reliability analysis) may help identify questions whose responses could potentially be biased by awareness rather than actual experiences. 

In OGE operations, insufficient sleep and changing shift pattern are the primary cause of fatigue [11]. Traditional work schedules are typically 7–14-day tour, with sequential 12-h shifts and shift changes. Evidence suggests that 12-h shift are not necessarily detrimental, but when coupled with extended workdays (i.e., beyond 4 days) and/or night work, there are many adverse effects on worker performance, safety and health [11]. As sleep and shift-related factors are a common source of fatigue, the FRAME survey has nine items to effectively assess these factors. A common practice in OGE operations is the use of a swing shift (i.e., switching from a night shift to a day shift or from a day to night shift). Swing shifts are used to allow workers to become adapted to day schedule for them to return home after a hitch [56]. It has been observed that workers can physiologically adapt to 12-h night shift but their internal circadian cycle is desynchronized for at least the first 4-5 days [57]. But those who start with a day-shift then switch to night shift do not adequately adapt [57]. The common practice of switching shifts in the middle of a hitch could potentially introduce several health and safety. The responses from sleep and shift-related questions could help to test effectiveness or implications of a swing shift. In addition to understanding the implications of swing swifts, responses on the shift and sleep-related questions could explain or allow for better interpretation of other responses for other questions within the FRAME survey. For example, if a worker has reported a low score (i.e., did not recover between shifts) to the item “*To what extent did you recover completely between shifts*”, it should be taken into account if they report a high score (i.e., found it extremely difficult to pay attention) to the item “*To what extent did you find it difficult to pay attention to someone, even when being spoken to directly.”* By understanding the cause and consequence of fatigue, effective mitigation strategies can be designed, which ultimately may reduce fatigue-related injuries and incidents. 

Valuable feedback has been received from employee focus groups regarding the survey content. It was suggested that a Spanish version of the FRAME survey would be highly beneficial. According to the BLS, 32.3% of workers within extraction are Hispanic or Latino, while the cross-industry average is merely 16.1% [58]. In 2011, 25% of OGE worker fatalities were Hispanic or Latino [53]. In addition to NIOSH identifying *“decrease-fatigue related injuries and fatalities in the oil and gas extraction industry”* as a top strategic goal for the Oil and Gas Extraction Program, *“reduce the incidence of injuries, illnesses, and fatalities among vulnerable workers in the oil and gas extraction industry such as contract workers, young and old workers, workers new to the industry, and immigrants”* has also been determined as a strategic goal. The addition of a Spanish version will allow for a greater number of workers to be properly assessed. However, future research would be needed to assess the readability of the FRAME survey with Spanish-speaking workers.

There are a few limitations that should be mentioned. First, the employee focus group was completed using only one onshore well-servicing company. However, the second Delphi Consensus Panel was composed of representatives from major energy companies. By using both small and major companies, considerations from different size companies were incorporated in the development of the FRAME survey. While subjective assessments only provide workers’ perception of their fatigue, due to the dynamic working environment of OGE operations, an industry-specific survey that is feasible, sustainable, and non-intrusive appears to be a viable approach to improving our understanding of fatigue. However, objective assessments beyond physiological measures need to be explored for OGE operations. Future research is required to test the reliability and validity of the FRAME survey in onshore and offshore OGE operations against traditional fatigue assessment methods such as performance tests and physiological monitoring. In addition to testing the reliability and validity of the FRAME survey, the scoring method should also be evaluated. A potential scoring method could be to sum the response for each dimension individually. The sum score of each dimension may identify what aspect of fatigue the workers are at the greatest risk, which could help to identify mitigation practices. Finally, while the FRAME survey provides worker perceptions on the degree to which fatigue impacts their physical and cognitive capabilities, future work could investigate coupling the survey with quantifiable fatigue sources (e.g., weather, rig activity—that implicates physical and cognitive workload, shift lengths/durations) to provide better decision support to supervisors and schedulers for effective and guided fatigue management practices. It is possible that, while the Delphi panel added question items, the panel members were biased by the nature of questions in the existing surveys—which were largely focused on identifying and quantifying symptoms rather than identifying the role of work factors.

## 5. Conclusions

Fatigue is a critical work risk factor in the oil and gas industry and one of the barriers to effective fatigue management is its assessment. The present study adopted a participatory mixed-method approach to develop a 26-item survey to conduct fatigue risk assessment and management in high-risk environments (FRAME survey), that assessed fatigue across four major dimensions, namely, sleep, shiftwork, physical, and mental fatigue. Because the FRAME survey was founded on occupational fatigue science and refined and tailored to the oil and gas industry, through rigorous industry stakeholder input, it will facilitate safer, effective, practical, and sustainable fatigue assessment and management efforts.

## Figures and Tables

**Table 1:** Participant Demographics.

	Delphi Panel 1 (n = 8)	Delphi Panel 2 (n = 5)	Worker Focus Group (n = 11)
**Gender**			
Male	6	4	100%
Female	2	1	
**Age (Years)**	38.57 (12.45)	38.40 (17.47)	44.36 (3.98)
**Race**			
American Indian	0%	0%	18.2%
Black or African American	0%	0%	9.1%
White	62.5%	60%	18.2%
More than one race	12.5%	0%	8.3%
Unknown or not reported	25%	30%	41.7%
**Ethnicity**			
Hispanic or Latino	12.5%	0%	54.6%
Not Hispanic or Latino	87.5%	100%	27.3%
Unknown or not reported	0%	0%	18.2%
**Education**			
Some High School	0%	0%	18.2%
HS Graduate or Equivalent	0%	0%	54.6%
Some College	100%	100%	9.1%
Unknown or not reported	0%	0%	18.2%
Experience (Years)	8.71 (11.22)	11.70 (11.29)	16.11 (4.36)

**Table 2:** Final Fatigue Inventory Questions.

Item	Administered		Dimension of Fatigue			
	Pre-Shift	Post-Shift	Physical	Mental	Sleep	Shift-Related
To what extent are you having to re-do or repeat tasks?	X	X	X	X	X	X
To what extent are you experiencing eye strain?	X	X		X	X	X
To what extent do your legs feel tired or heavy?	X	X	X			
To what extent are your joints (e.g., knee or elbow) feeling achy or stiff?	X	X	X			
To what extent are you experiencing stiff muscles?	X	X	X			
To what extent are you having trouble concentrating?	X	X		X		
To what extent are you having trouble paying attention (e.g., like during meetings or briefs)?	X	X		X		
To what extent has your sleepiness interfered with your work?	X	X			X	X
To what extent are you still tired even after waking up from sleep?	X				X	X
To what extent did you recover between shifts?	X				X	X
To what extent did you have trouble sleeping?	X				X	X
To what extent did you feel exhausted?		X	X	X	X	X
To what extent did you experience cramps in your muscles?		X	X			
To what extent did you experience rapid heartbeats?		X	X			
To what extent did your legs feel numb?		X	X			
To what extent did your arms, hands, and/or fingers feel numb?		X	X			
To what extent did fatigue interferes with your physical functioning.		X	X			
To what extend did fatigue interferes with your mental functioning.		X		X		
To what extent did you experience blurred or distorted vision?		X		X		
To what extent did you daydream during work duties?		X		X		
To what extent did you have trouble remembering work-related things (i.e., instructions or procedures)?		X		X		
To what extent did you find it difficult to pay attention to someone, even when you were being spoken to directly?		X		X		
To what extent did you find it easy to keep track of everything was going on around you?		X		X		
To what extent did you have trouble getting back into work after an interruption?		X		X		
To what extent did you experience difficulty staying awake during work?		X			X	X
To what extent did you feel drowsy during your shift?		X			X	X
Total	11	20	10	12	9	9
